# The Law’s Charlatanry

**Published:** 1881-01

**Authors:** Ausburn Towner


### THE LAW'S CHARLATANRY.

AUSBURN TOWNER.

### II.

In the BISTOURY for October, I laid before its readers some illustrations of the slavish adherence in the practice of the law to precedents, and attempted to show how it continually worked evil and injustice. These illustrations, as I observed, were not sought for nor gleaned, but, as I might say, presented themselves to me in the desultory reading of the newspapers. One month's work would gather from the current business of the courts of New York city alone, more than enough absurdities, and questions offensive to the ordinary intelligence, than would be sufficient to fill volumes twice as large as the Revised Statutes of the State of New York.

A litigated case, from the service of the summons to the verdict of the jury, except for the interest involved, has come to be not at all above the dignity of a base ball match or a horse race, with its termination just as much the subject of a wager, just as much the object of chance or skill. To a disinterested person watching its progress, the questions of right or justice do not seem to enter at all into the game, and even if they crop up now and then, they are relentlessly jammed into the background. The object aimed at seems to be: What is the Law, or who is the shrewdest lawyer, the most skillful dodger, the most impressive advocate? A set of cast iron rules and decisions like those of the National Base Ball Association are set up and every question that arises must be squeezed and pinched and tortured from its surrounding to fit them, rather than an end should be reached founded on the present facts. It gives the impression of being simply a game, in which tricks are played of a character, that if similar ones were only attempted among the same gentlemen in a hand at poker, the offender would be banished from decent society, if he was not thrown head foremost out of a window; a game where the stakes may be enormous, and which each side is playing to win, right or wrong. All of the costly, useless and foolish machinery of our courts, in the way of appellate jurisdiction arises solely from this adherence to the idea of precedents. Cases are never sent up unless where there may be newly discovered evidence, no any question that goes to the merits of the matter in dispute. The Law takes them up and not the facts. It is claimed that some error has been committed. Of what kind? Never as to the substance; always to the form. One of the lawyers may have asked a question, no matter how necessary to the elucidation of the truth, in an improper way, or at least decided to be improper, years and years ago, and the Judge permitted it to be answered; or the Judge in his charge, said something that a long time ago had been said by some one, he had no right to say, and back the case is sent for a new trial, redoubling the expense to suitors, wearing out their patience and casting ridicule on the whole machinery of the Law. Some of these alleged errors are as reasonable in their foundation as it would be to grant a new trial because one of the lawyers wore check trousers or the judge was bald-headed.

This slavish adherence to precedent complicates, muddles and disfigures the Law; it throws serious obstacles in the way of justice; shields the guilty from the penalty justly due their crimes and protects the unscrupulous, wealthy and greedy in the possession of their ill-gotten gains, for the innocent and honest should not need to hide behind subterfuges and technicalities; it degrades jurisprudence to the level of thimble-rigging and the sweat-board; makes it possible for a contest like the Vanderbilt will case to sully the records of the courts; gives rise to such sneering truisms as "the uncertainties of the law," and passes them into proverbs; makes a profession that should be reputable and decent, the scoff and bye-word of reproach of the world; permits opera singers* to advertise themselves at its expense, and carries a suit from one court to another, increasing the expense in geometrical proportion, oftentimes impoverishing the suitor, and if ever reaching justice oftener brings it too late for any good.

The more and the deeper one goes into this subject, the more obnoxious abuses are met at every step. It is not definitely known to the general public, although there is a dim notion

*Whether Jennie R. Smith and Covert D. Bennett were guilty or innocent of the murder of the Jersey city police officer, the husband of the first named, it is a great shame to the jurisprudence of our country that except for the want of a little money that Miss Emma Abbott obtained by her own personal exertions, they would have been debarred the common privilege of a fair and decent trial.

of it to be gathered from the constant litigation that it entails, of the enormous quantity of rot that has grown up called "The Law in regard to Insurance;" that there have been books after books written upon the subject and that there are men, who, after a life time of study spent upon it, have made it a specialty in their profession. The Law in regard to Insurance indeed! To the ordinary apprehension, it should reduce itself to the very simplest of contracts. Why should there be any except that when a man bets $40 or $50, as the case may be, that his house or his manufactory will burn in a certain specified time, against $2,000, or $10,000 that they will not burn, and they *do* burn, that the Insurance company shall pay its bet? It seems to me that in such a manner an honest and an honorable man or men would reason. The Law makes it otherwise and has so buried a plain agreement in meshes of uncertainty and strange decisions that it has come to be the received opinion, that a company's only policy, is first, to get the insured's premium, and then see how it can escape the penalty of a loss, if one occurs, or, if it must pay, to get the sum down to the lowest possible figure. In this policy, at least, the Law of Insurance aids them to an extent that one would hardly believe, unless he examined the subject carefully.

Nor is the Law of Insurance the only branch of jurisprudence that the adherence to precedent has degraded to a catchpenny device. The Law of contracts, of railroads, of hotels and boarding houses, of common carriers, of marriage and divorce of every interest between man and man, has been tinkered and patched over and pinched down to its injury and discredit. The Law as it has come to be administered, touches nothing that it does not mystify and befog, until the straight path has become more entangled and obscure than Ariadne's labyrinth. The lawyers, of course, understand this and assist it to their best ability. It is money in their pockets, reputation for themselves and subsistence and position for their families.†

Sometimes, it is a great deal more, as witness the immense fees that some of our legal gentlemen manage to secure and it is hard to tell how it is possible for them to render any sort of an equivalent in service therefor; and that one in New York who recently settled a solvent estate of $500,000 and all the widow and children had to show for it was a bill that he rendered her for services amounting to a good round sum!

There is no man, who has mingled, as Americans do with the world, who cannot recall instance upon instance of injury, harm and wrong worked by the Law as it is now administered; three to one where it has been the parent of any good. I suppose these things do not meet with the condemnation they deserve because they are so common that they have ceased to attract notice. There will be a terrible awakening one of these days. Look at the jury system, as at present regulated. It is a relic of our half savage ancestors in the island of Britain; something that civilization out grew years ago, but it has precedent to sustain it. It is beneath the dignity of a side show to a third rate circus and must make every justice, who has a spark of intelligence about him blush for his position and his kind. Look at the insulting and tyrannical manner in which lawyers are permitted to treat witnessess,‡ a manner in comparison with

†"Mr. Greeley," said Partridge, "this is Mr. Denslow, a young attorney." Greeley uttered a short grunt of recognition, but did not even look around. I, embarrassed, shrank away to one corner and took a chair. He went around the room, looking at pictures and what not, and in about five minutes, when his back was turned to me, and I thought he had forgotten me, he suddenly, without looking at me, said; "Hem! So, you're an attorney are you?" I confessed it. "I hate lawyers," he exclaimed emphatically. "I hate lawyers; they do more mischief than their heads are worth."

"I suppose they are necessary evils," I suggested deprecatingly.

"Wholly unnecessary," he insisted.

"I suppose you will acknowledge," I said, "that they promote good order and remove impediments to good government."

"Just the contrary, just the contrary," he squeaked in his odd falsetto. "They cause disorder and are the chief obstacles to good government."

I thought the man crazy. "Perhaps you will tell me," I suggested, "how debts would be collected without lawyers."

"Don't want 'em collccted! don't want 'em collected!" he squeaked." "If A lets B have his property without payment, I don't see why C. D. E. and F. and all the rest of the alphabet shouid be called in to serve as police to get it back! No debt should be collected by law. Its monstrous! Let a man trust another man at his own risk. Even a gambler pays his debts that he isn't legally obliged to pay and calls them debts of honor; but men will put their property out of their hands to prevent the legal collection of their grocery bills. Abolish all laws for the collection of debts and that would abolish most of your lawyers—good riddance.—*N. Y. Correspondence Indianapolis Journal.*

‡A short time ago, a jury was sitting on a case in Southern Colorado. A woman took the stand and was somewhat unceremoniously treated by the lawyers. At last, a particularly dry and caustic fellow began to practice on her with the cross-examination. Now among the jurymen was one gentleman who had been drawn with apprehension and fear of consequences. He was a free, wild miner, with no more idea of the law than a buffalo. He twitched uneasily while the woman was under examination and could be kept still only by being provoked into a whispered conversation with the foreman. As the cross-examintion reached a particular point, he astonished the court, by jumping up, thrusting his hand into his hip pocket and exclaiming to the lawyer: "Hi! thar! You, sir!" This from a juryman, brought every eye in wonder and amazement upon him. He heeded nothing and continued: "Jack McCabe won't 'low no man to talk to a woman in that shape; not while he's 'round." The Judge, of course, rebuked honest Jack, who sank into his seat, embarrassed, but mad. The lawyer from his vantage ground, turning upon him with withering scorn, began bombastically: "Of what weight with me is the opinion of an ignorant jurymen? I—" "That's what I thought," interrupted Jack, as with one bound, he cleared the rail and wound himself around which the language and behavior of a drunken bar-room loafer become the most polished decoum and the most cultured politeness. What is it that so transforms an otherwise intelligent and gentle hearted man into a brute and a coward, if perchance it becomes his duty to cross-examine a witness? I have seen such transformations and marvelled at them. Look at the fact that in New York city, by these technicalities and decisions, the statute law abolishing imprisonment for debt is utterly a dead letter, or that still more outrageous fact in the city named, that witnesses may be imprisoned, or as it is, with an assumption of delicacy put, committed to the House of Detention, while the criminal against whom they are expected to testify is permitted to be at large.¶

Look at the manner in which most of the law officers of the county or state, seem to misinterpret their duties. Did the reader never notice the eagerness that a District Attorney manifests to convict an accused person as though the number of convictions that he secured, right or wrong, measured his usefulness as a public servant? It has always seemed to me that his desire was, rather that some one should be convicted than that the guilty one should be discovered. Is it not as much a part of his duty to ascertain the truth as it is to convict some one *anyhow*? Yet he will quibble and dodge and invent and depend upon tricks and technicalities and subterfuges, rather than place himself squarely and firmly on what he should believe to be the truth that is in his possession. Perhaps the Law forces him into this degrading position, and he is obliged to fight with the weapon it has furnished him, to set forth the truth. Perhaps there are so many loop-holes, so many corners, so much protection for crime, that unless he did so fight even the guilty might escape.** So much the more for the law then, and so much the more does it cry out for a radical change.

The inquiry naturally arises: Where is the remedy? What can be done to relieve the public of such a burden as the Law has become? To overturn the present system would be almost like destroying society and beginning all over again, it is so like an old sore that has attached itself to the very vitals. It seems to me that if a change could ever be wrought, we would look back upon the system as we now look back upon slavery, hardly believing that this free (?) country was ever cursed with such a monstrosity.

One thing cannot be denied. There will never be a change for the better as long as lawyers are permitted to form the larger portion of our legislative or governing bodies. As men, the members of the profession are neither better nor worse than the average man. It is to their interest to draw laws loosely or to have them unacted upon, and as long as human nature is as it is, they will do so, if they have the power.

One other conclusion. Courts should be so constructed, and it does not seem to me to be impossible, that each case or each question should be decided for and by itself and its own surroundings without regard to what has gone before or what is to come after. This would sweep out of suits-at-law all the rubbish that now encumbers them, and that really has no bearing upon the facts and the truth to be got at, would abolish much expensive and useless machinery and substitute for it a simpler system. It might drive some otherwise excellent gentlemen to seek other means of subsistence but the great mass of men would be the gainers. It should be that one who has experienced a grievance or suffered a wrong, might feel, that, as he was able to establish his facts, so would he be sure to obtain his remedy and not, that howsoever clear might be his case, some technicality or absurd decision rendered under an entirely different state of affairs, would thwart the right, or at the very best, his struggle would be an uncertain one. The Law and the practice should and can be so thoroughly perfected and simplified that they will be as easily understood and as incontrovertible as the multiplication table. The first movement toward such a desirable consummation will be a disregarding of ancient and musty prejudices and precedents, and the relegation of all the miscalled learning packed away in reports and text books, to the obscurity and contempt that belong to those other equally important elements of human prothe lawyer. Before a constable could reach the pair and arrest the assailant, Jack had macerated the lawyer so that he was obliged to give up the case and go home on a shutter. More than one listener has seen the time when he would go a hundred on such human nature as Jack displayed to one on the law of the lawyer.

¶This anomalous proceeding, a satire on our boasted privileges, is of almost daily occurrence in New York city.

**I have heard it set forth as an assumed reason for these technicalities that they are safeguards and protections for the innocent! Is it not a little strange that they never come to the surface only when notorious criminals and outlaws are on trial and that these persons are the only ones ever known to take advantage of them! The assumed reason seems to be a very large handful of dust that legal gentlemen have been in the habit of throwing into the eyes of the public for generations.

gress, good enough as stepping stones, alchemy, astrology, perpetual motion and the elixir vitæ. The shibboleth, "Judge Blank held so-and-so in the great case of Doe vs. Stiles, deserves to become as obsolete as Abracadabra."

